# Genomic Analysis of Hexokinase Genes in Foxtail Millet (*Setaria italica*): Haplotypes and Expression Patterns Under Abiotic Stresses

**DOI:** 10.3390/ijms26051962

**Published:** 2025-02-24

**Authors:** Wei Zhou, Xiaoning Cao, Hangyu Li, Xiaokuo Cui, Xianmin Diao, Zhijun Qiao

**Affiliations:** 1 College of Agriculture, Shanxi Agricultural University, Taigu, Jinzhong 030801, China; nx06114@163.com (W.Z.); lhytwofour@foxmail.com (H.L.); 2 Shanxi Institute for Functional Food, Shanxi Agricultural University, Taiyuan 030031, China; 3 Center for Agricultural Genetic Resources Research, Shanxi Agricultural University, Taiyuan 030031, China; caoxiaoning2008@163.com; 4 Key Laboratory of Crop Genetic Resources and Germplasm Development in Loess Plateau, Ministry of Agriculture and Rural Affairs, Taiyuan 030031, China; 5 Institute of Crop Sciences, Chinese Academy of Agricultural Sciences, Beijing 100081, China; cxk2823466386@163.com

**Keywords:** *HXK* genes, abiotic stress, subcellular localization, haplotype analysis, foxtail millet

## Abstract

Hexokinases (HXKs) in plants are multifunctional enzymes that not only phosphorylate hexose but also function as glucose sensors, integrating nutrient, light, and hormone signaling networks to regulate cell metabolism and signaling pathways, thereby controlling growth and development in response to environmental changes. To date, limited information is available regarding the *HXKs* of foxtail millet (*Setaria italica* L.). In this study, six *HXK* genes were identified and characterized in foxtail millet. Phylogenetic analysis revealed that the foxtail millet hexokinases were classified into three subfamilies, corresponding to the two types (B-type and C-type) of hexokinases in plants. Gene structure and conserved motif analysis showed that the *SiHXKs* exhibited varying numbers of introns and exons, with proteins in each subfamily showing similar motif organization. Evolutionary divergence analysis indicated that the foxtail millet *HXK* and green foxtail *HXK* genes families underwent both positive and negative selection and experienced a large-scale duplication event approximately 1.18–154.84 million years ago. Expression analysis revealed that these genes are widely expressed in roots, stems, leaves, panicles, anthers, and seeds, with most genes showing significantly increased expression in roots under abiotic stress conditions, including 20% PEG 6000 (drought stress), 200 μmol/L NaCl (salt stress), and 1 μmol/L BR (brassinosteroid-mediated stress response). These results suggest that these genes may play a pivotal role in enhancing stress tolerance. Subcellular localization assay showed that *SiHXK5* and *SiHXK6* were predominantly localized in mitochondria. Haplotype analysis revealed that SiHXK3-H1 was associated with higher plant height and grain yield. These findings provide valuable insights into the functional characteristics of *HXK* genes, especially in the context of marker-assisted selection and the pyramiding of advantageous haplotypes in foxtail millet breeding programs.

## 1. Introduction

Carbohydrate metabolism is crucial for plant growth, development, and seed yield by providing the necessary energy and materials for synthesizing storage compounds, proteins, and nucleic acids [1]. In higher plants, sugars as the primary carbon source are essential for the biosynthesis of various cellular compounds, energy generation, and osmotic processes [2]. Glucose and other hexoses play crucial roles as universal carbon sources and sugar signaling molecules, regulating a wide range of biological processes, including carbohydrate metabolism, protein accumulation, and sucrose transport, in higher plants [3]. HXK is a glycolytic regulatory enzyme in plants that catalyzes the irreversible phosphorylation of D-hexoses at the sixth carbon using ATP-Mg^2+^ as a phosphate donor, thus converting glucose and other hexoses into hexose 6-phosphates [4].

*HXK* family members can be localized in the cytosol, mitochondria, nucleus, or chloroplast, depending on the presence of anchor or signal peptides in their N-terminal sequence [5]. They are divided into two main types (type A and type B) and two rare types (type C and type D) [6]. Types A and B are the primary hexokinases responsible for maintaining fundamental cellular metabolism and energy homeostasis, while also playing crucial roles in stress signaling pathways. In contrast, types C and D are relatively rare and less well characterized, but they are thought to provide specialized functions in response to specific stress conditions or in particular tissues or organs. Type A *HXKs*, such as *AtHXK3* [7], *OsHXK4* [8], and *NtHXK2* [9], have a 30-amino acid hydrophobic sequence at the N-terminus that encodes a chloroplast signal, directing them to the chloroplast. Type B *HXKs*, including *AtHXK1*, *AtHXK12*, *LeHXK1*, *LeHXK2*, and *LeHXK3* [7,10], feature a highly hydrophobic transmembrane helix composed of 24 amino acids, which anchors the protein to the mitochondria. Some type B *HXKs* may also localize to the nucleus due to a nuclear localization signal near the membrane-anchoring domain. Type C *HXKs*, lacking signal peptides or membrane-anchoring domains, are mainly found in the cytosol and are predominantly present in monocots and mosses [5,11]. Type D *HXKs* are mitochondrial proteins with a unique mitochondrial-anchoring peptide distinct from that of type B, found exclusively in mosses. Sequence analysis has revealed several conserved domains in hexokinases, such as phosphate 1, phosphate 2, connect 1, connect 2, and the adenosine phosphate binding region, which are instrumental in identifying and predicting the functions of *HXK* genes [3].

*HXKs* serve as essential switches for sugar signal sensing and transduction [4]. *HXKs* in plants, particularly *AtHXK1* in Arabidopsis, act as critical glucose sensors, regulating various biological processes independently of their enzymatic activity in phosphorylating glucose [7,12]. In *Arabidopsis thaliana*, these enzymes modulate photosynthesis by sensing glucose levels and transporting sugar signals from the chloroplast to the nucleus, thereby activating photosynthetic genes and coordinating photosynthesis with transpiration [13,14]. Mutants of *AtHXK1* show altered growth patterns, delayed flowering and senescence, and changes in hormone sensitivity, highlighting the comprehensive role of *HXKs* in plant growth and development [14]. The suppression of mitochondria-associated *NtHXK1* in tobacco plants results in inhibited growth and increased starch and glucose accumulation, underscoring the critical role of *NtHXK1* in sugar signaling, particularly in regulating starch turnover, glycolytic activity, and overall plant growth [15]. Transgenic rice plants overexpressing *OsHXK5* or *OsHXK6* exhibit reduced photosynthetic gene expression under glucose treatment, suggesting that these hexokinases function as glucose sensors by localizing to both mitochondria and nuclei, thereby enhancing the glucose-dependent repression of specific genes and influencing photosynthesis, gene expression, and plant growth [16].

Moreover, *HXKs* play a crucial role in regulating stomatal behavior, thereby enhancing abiotic stress resistance by modulating guard cell responses. Research shows that sucrose, processed via *HXK* and abscisic acid, effectively triggers stomatal closure, conserving water and minerals during periods of high sucrose production, thereby reducing water loss [17]. This forms a feedback loop where excess sucrose beyond phloem capacity initiates stomatal closure through *HXK*, crucial for coordinating CO_2_ uptake, sugar production, and water conservation, which are essential for managing environmental stress [18,19]. Furthermore, *HXK* influences broader physiological processes, such as photosynthesis and nutrient absorption in both mesophyll and guard cells, essential for optimal plant growth and survival under drought conditions. In Populus, *PdHXK1* interacts with the GATA family transcription factor, *PdGNC*, significantly enhancing drought resistance by increasing *HXK* activity, which not only improves sugar metabolism but also stimulates the production of signaling molecules like nitric oxide and hydrogen peroxide, reducing stomatal opening and minimizing water loss [20]. Similarly, in Arabidopsis, plants engineered with the Glycine max *HXK2* (*GmHXK2*) gene exhibit improved salt stress tolerance, while those with silenced *GmHXK2* show decreased salt tolerance gene expression, increasing their vulnerability to salt stress [21]. The ectopic expression of the Prunus *HXK3* gene in Arabidopsis notably boosts tolerance to both salt and drought stresses [22]. Additionally, a positive correlation has been observed between the *GmHXK15* gene and *HXK* activity, where the overexpression of *GmHXK15* in soybean hairy roots significantly enhances their alkali stress resistance [23]. In maize, the expression of *ZmHXK5* and *ZmHXK6* were significantly increased under cold (4 °C), salt (200 mM NaCl), and drought stress (20% PEG 6000) [24].

Foxtail millet (*Setaria italica*) is distinguished by its small genome, short growth cycle, and its robust drought and poor soil tolerance, establishing it as a novel model crop [25]. *HXKs* have been extensively studied in Arabidopsis [7], rice [8], sorghum [26], and various plant species [1,23,24,27]. However, research on *HXKs* in foxtail millet remains limited, particularly regarding their roles in abiotic stress responses. In this study, the *HXK* gene family in foxtail millet was comprehensively identified and characterized using a combination of bioinformatics and experimental approaches. The expression patterns of this gene family across different growth stages of millet were systematically analyzed, their transcriptional responses under abiotic stress were investigated, and subcellular localization studies were performed. Additionally, a comprehensive analysis of haplotype variations and their genetic effects was conducted, providing a solid foundation for the functional characterization and future breeding applications of the *HXK* gene family.

## 2. Results

### 2.1. Identification and Characterization of HXKs in Foxtail Millet

To identify *HXK* genes in foxtail millet, two approaches were employed: a comparison of six HXK proteins from Arabidopsis with foxtail millet protein sequences using BLASTp (version 2.10.1+), and a genome-wide search of foxtail millet using the HMM model (PF03055). These analyses collectively resulted in the identification of six *HXK* genes (*SiHXK1*–*SiHXK6*) (Table S1). The CDS lengths spanned from 1400 bp for *SiHXK1* to 1543 bp for *SiHXK2*, while the corresponding protein lengths ranged from 461 amino acids (aa) for *SiHXK1* to 508 aa for *SiHXK2*. The molecular weights of these proteins varied from 50.05213 kDa for *SiHXK1* to 54.85187 kDa for *SiHXK2*. Detailed information about each *SiHXK*, including additional properties such as isoelectric point (pI) and genomic locations, is provided in Table 1.

### 2.2. Phylogenetic Relationships and Multiple Alignments of HXKs in Foxtail Millet

**Figure 1:**
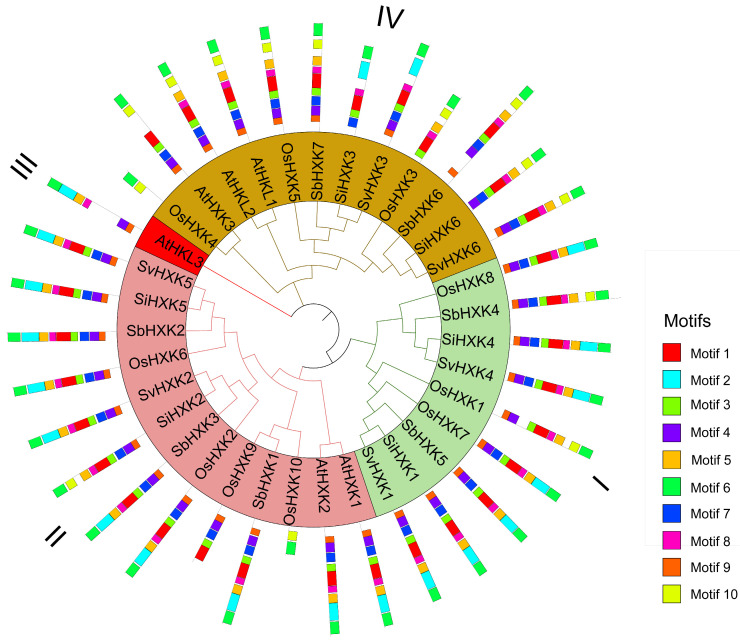
Phylogenetic tree and motif composition of *HXK* proteins from foxtail millet, Arabidopsis, rice, sorghum, and green foxtail millet. Different subfamilies are represented by different colors. Motif sequences are listed in Table S2.

Previous research has documented the presence of *HXK* genes across various plant species [7,8,23,24]. To investigate the evolutionary relationships between *SiHXKs* and *HXKs* from other plant species, 35 full-length HXK protein sequences were analyzed (Table S1). This set included six sequences from Arabidopsis, ten from rice, six from green foxtail, seven from sorghum, and six from foxtail millet. Phylogenetic analysis revealed that the *HXK* genes were broadly categorized into four subfamilies, with *SiHXK* members distributed across three of these subfamilies (Figure 1). All the *SiHXKs* showed high sequence similarity to *SbHXKs* and *OsHXKs*. *SiHXK1* and *SiHXK4* were clustered into class I, class II contained *SiHXK2* and *SiHXK5*, and class IV consisted of *SiHXK3* and *SiHXK6*. *SiHXK1* and *SiHXK4* were clustered into class I and were closely related to three type-C *OsHXKs*—*OsHXK1*, *OsHXK7*, and *OsHXK8*—on the same evolutionary branch. Notably, these genes neither contained chloroplast transit peptides or membrane anchors. Conversely, *SiHXK2*, *SiHXK3*, *SiHXK5*, and *SiHXK6* align with other type-B *OsHXKs*, which contained a membrane anchor domain, distinctly localized in the mitochondria.

**Figure 2:**
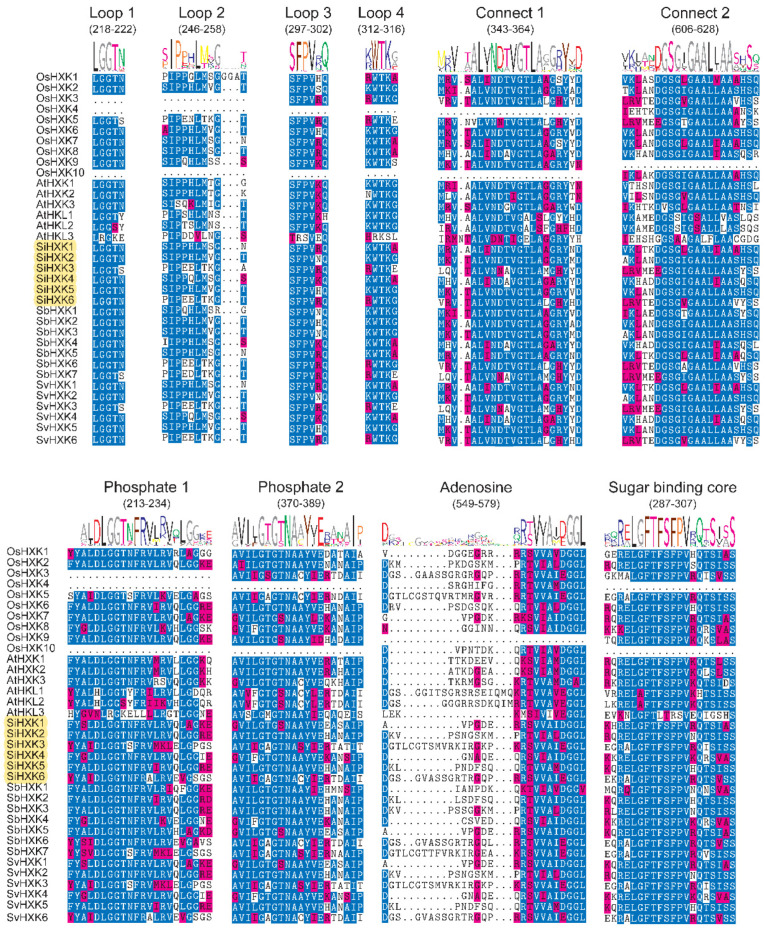
Multiple alignments of *HXKs* amino acid sequences in five species. The sequences were aligned and visualized using the msa package in R [28]. Blue represents conserved sequences, red indicates non-conserved regions, and the yellow shadow represents the six *HXK* genes in foxtail millet. The inset image at the top represents ten conserved sequences, which were based on prior characterizations of *HXK2* in *Saccharomyces cerevisiae*. The height of each letter in the upper section of the panel represents the specific amino acid conservation in each motif.

To further analyze the SiHXK proteins, their sequences were aligned using the R package msa [28]. The identification of conserved sequences was based on prior characterizations of *HXK2* in *Saccharomyces cerevisiae*, as detailed in studies [29,30,31]. All SiHXK proteins retained conserved domains (Loop 1, Loop 2, Loop 3, Loop 4, connect 1, connect 2, phosphate 1, phosphate 2, adenosine, and sugar binding core) and exhibited complete loop and connection structures, underscoring their structural integrity and potential functionality in carbohydrate metabolism (Figure 2). These results suggest an evolutionary divergence among *SiHXKs* and also the functional similarity of *SiHXK* members in the same class.

### 2.3. Gene Structure Analysis of Foxtail Millet HXK Genes

**Figure 3:**
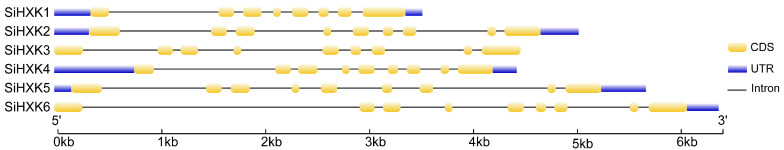
Gene structure of foxtail millet *HXKs*. Gene structure of *SiHXK* genes, including introns, UTRs, and CDSs. The scale bar at the bottom represents the length of genes. UTR: untranslated region; CDS: coding sequence.

The sequence structure analysis of six foxtail millet *HXK* genes reveals that *SiHXK1* had seven introns and was the shortest gene, while the other five genes each had eight introns. *SiHXK6* was the longest gene. Four genes (*SiHXK1*, *SiHXK2*, *SiHXK4*, and *SiHXK5*) contained both 5′ and 3′ untranslated regions (UTRs). In contrast, SiHXK6 only contained a 3′ UTR, and *SiHXK3* lacked UTRs altogether (Figure 3).

Further analysis of the conserved motifs shows that *SiHXK1* and *SiHXK4* did not contain motif 9, while the other four foxtail millet *HXK* genes included all ten motifs (Figure 1). The conserved motifs of *SiHXK* members in the same group support the reliability of the group classifications by the phylogenetic analysis.

### 2.4. Evolutionary Patterns of Foxtail Millet, Rice, Sorghum, and Green Foxtail Millet

In this study, selective pressures on *HXK* genes were investigated by comparing the homologous genes of foxtail millet with those of rice, sorghum, and green foxtail. The analysis revealed five homologous gene pairs between foxtail millet and rice, one pair between foxtail millet and sorghum, and ten pairs between foxtail millet and green foxtail (Table 2). By calculating the non-synonymous (Ka) and synonymous (Ks) substitution rates and their ratios, it was inferred that gene duplication events between foxtail millet and rice occurred approximately 37.31 to 336.15 million years ago (MYA), those between foxtail millet and sorghum around 330 MYA, and events between foxtail millet and green foxtail likely occurred between 1.18 and 154.84 MYA, with the most recent divergence at about 1.1 MYA. These findings offer critical insights into the selective pressures and evolutionary trajectories that have shaped the *HXK* gene family.

### 2.5. Cis-Acting Element Variations of HXK Members in Foxtail Millet

Cis-acting elements were predicted within the 2 kb upstream region of the start codon of *SiHXK* genes (Figure S1). The analysis revealed a variety of regulatory elements, including meristem expression (CAT-box) and seed-specific (RY-element) elements, as well as hormone-responsive elements such as ABRE (abscisic acid), CGTCA-motif (methyl jasmonate), ERE (ethylene), GARE-motif (gibberellin), MBS (flavonoid), and TCA-element (salicylic acid). Additionally, multiple abiotic stress-responsive elements were identified, including those for anaerobic conditions (ARE and GC-motif), drought (MBS and DRE), low temperature (LTR), and salt (TCA), and responses to heat, osmotic stress, low pH, and nutrient deficiency (STRE). Variations in the presence and number of these elements among different *HXK* genes suggest diverse regulatory mechanisms that may underlie their roles in plant growth, development, and stress responses. 

### 2.6. Expression of Foxtail Millet HXKs in Various Tissues at Different Growth and Development Stages

**Figure 4:**
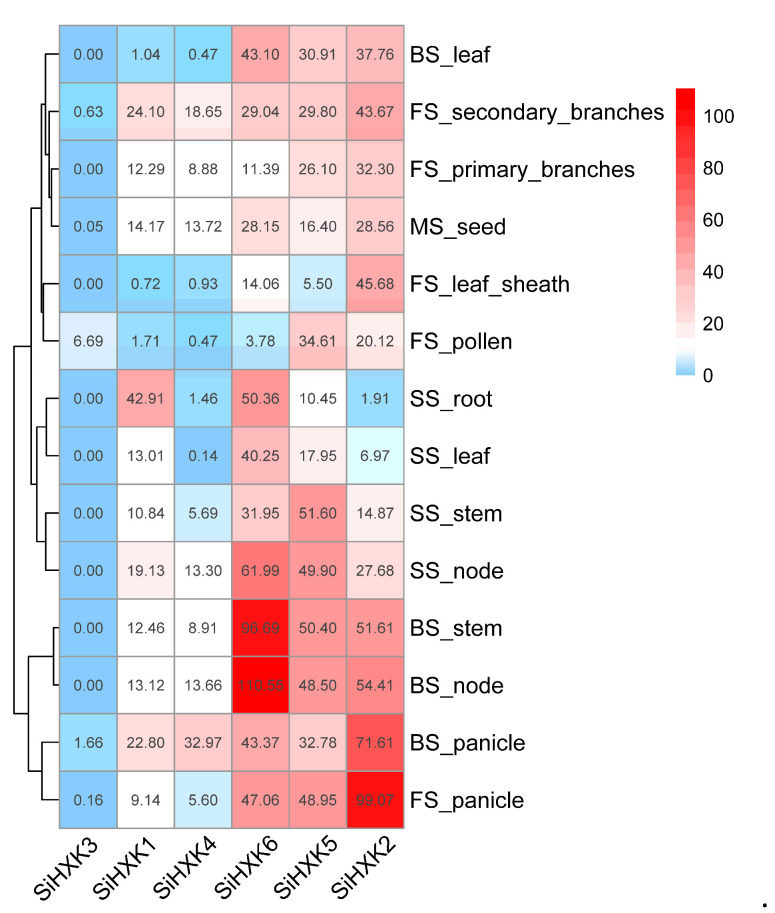
Transcription profile of *SiHXKs* in various tissues at different growth and development stages of foxtail millet. Transcription data were generated by RNA-seq sequencing and were calculated as TPM. BS: booting stage; SS: shooting stage; FS: flowering stage; and MS: mature stage.

To further uncover the possible functions of *SiHXKs*, we examined the expression patterns of *SiHXKs* in various tissues of Yugu1 at different growth and development stages using RNA-seq data from the Setaria-db public database [32]. The results indicate that different genes exhibited distinct expression patterns under different development stages and tissue conditions (Figure 4). *SiHXK2*, *SiHXK5*, and *SiHXK6* showed high expression levels across all stages and tissues, manifesting multi-functions of these genes. In addition, the expression level of *SiHXK2* was exceptionally high in the panicle of booting stage and flowering stage, suggesting that *SiHXK2* might be involved in panicle differentiation and development. *SiHXK6* had remarkably high expression in the nodes and stems of the booting stage, suggesting it might function in regulating cell proliferation and the elongation of stems. *SiHXK1* showed higher expression in the root of shooting stage, indicating that it might be involved in root development. The expression of *SiHXK4* was high in the panicle of booting stage, suggesting that it might be involved in regulating panicle differentiation and development. *SiHXK3* barely expressed in all tissues, indicating that it had little or only a minor effect on foxtail millet growth and development.

### 2.7. Abiotic Stress Response of HXKs Genes in Foxtail Millet

**Figure 5:**
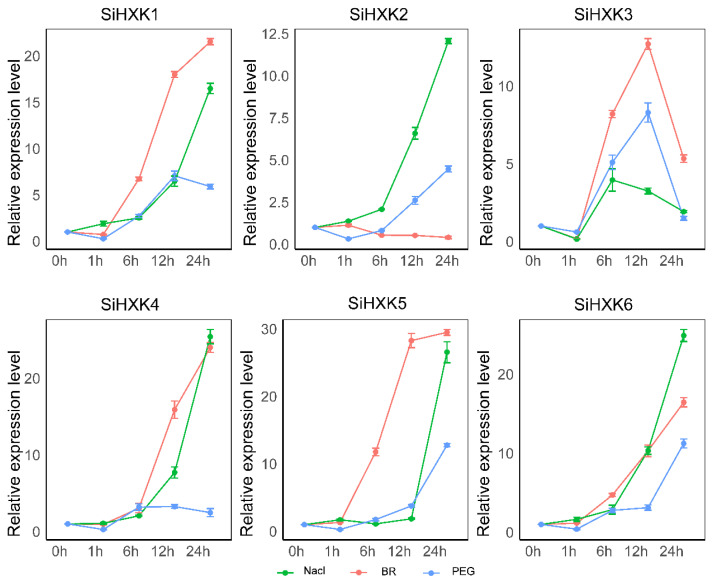
Expression pattern of *SiHXKs* in response to abiotic stress. Three abiotic stress treatments (20% PEG 6000, 200 µmol/L NaCl, and 1 µmol/L BR) were applied and root samples were collected at 0 h, 1 h, 6 h, 12 h, and 24 h after treatment. Relative transcript levels were normalized to *SiCULLIN*. Error bar represents SD values of three biological replicates.

To investigate the response of *SiHXKs* to various abiotic stresses, we examined the expression patterns of *SiHXKs* in the roots of seedlings subjected to treatment with 20% PEG 6000, 1 µmol/L BR, and 200 µmol/L NaCl [7] (Figure 5). The results demonstrate that all six *SiHXK* genes exhibit differential expression patterns in response to various abiotic stress treatments. High-salinity treatment was used to determine the possible roles of *SiHXKs* under salt stress. The transcript levels of *SiHXK1/2/4/6* were rapidly upregulated after NaCl treatment; *SiHXK1* expression was basically unaffected during the first 12 h, and then sharply increased from 12 h to 24 h; the expression level of *SiHXK3* was slightly upregulated during the first 1 h, and upregulated with the peak at 6 h, and then downregulated to the level before treatment. Under BR treatment, the transcripts of *SiHXK4/5/6* were significantly upregulated in 24 h; the expression levels of *SiHXK1* and *SiHXK3* slightly declined then rapidly increased, with the peak at 12 h for *SiHXK3*; *SiHXK2* expression was slightly decreased throughout the 24 h after BR treatment. A PEG 6000 simulation was performed to explore the potential roles of *SiHXKs* in response to environmental drought stress. The expression levels of all six *SiHXKs* were slightly decreased during the first 1 h; and then *SiHXK2*, *SiHXK5*, and *SiHXK6* were upregulated; *SiHXK1* and *SiHXK3* increased then decreased with the peak at 12 h after treatment; *SiHXK1* was slightly upregulated and then maintained a stable level. These results underscore the potential functional diversity of *SiHXK* genes in foxtail millet’s adaptation to various abiotic stress conditions.

### 2.8. Subcellular Localization of HXK Proteins in Foxtail Millet

Previous research has shown that the N-terminal trans-membrane peptide directs the HXKs to the mitochondria [4,12]. In the current study, subcellular localization assays were performed using GFP-fused *SiHXK5* and *SiHXK6* and pFAy-mCherry as a mitochondria marker. SiHXKs-GFP and pFAy-mCherry were coexpressed in Nicotiana benthamiana cells. The results showed that SiHXKs-GFP colocalized with the mitochondria marker, suggesting that *SiHXKs* has a molecular function in mitochondria. This confirms the mitochondrial localization of HXK family proteins in foxtail millet.

### 2.9. Analysis of Genetic Polymorphism in the Coding Sequences of Foxtail Millet HXK Genes

To assess the effect of variations in *SiHXK* genes on the agronomic traits of foxtail millet, we analyzed the coding region variants of *SiHXKs* using 1844 accessions published previously [33]. For *SiHXK1*, ten variations were found, including six synonymous mutations: T1335C (Gly445Gly), C1044T (Asp348Asp), T987C (Ser329Ser), T861A (Ser287Ser), G177C (Thr59Thr), and T90C (Ala30Ala), and four missense mutations: A1237G (Thr413Ala), G565C (Asp189His), T411A (His137Gln), and T374C (Val125Ala). These resulted in twelve haplotypes, with the major ones being H1 (1152), H2 (351), H3 (121), H4 (77), H5 (30), and H6 (17), and six rare haplotypes (frequencies less than 5). *SiHXK2* had five synonymous mutations in its exon region: C1260T (Ala420Ala), T1038C (Asp346Asp), C969T (Tyr323Tyr), T756C (Val255Val), and C207T (Asp69Asp), forming four haplotypes: H1 (1017), H2 (341), H3 (250), and H4 (175). *SiHXK3* exhibited eight variant types in its exon region, including six synonymous mutations: C1467T (Leu489Leu), T1209C (Ala403Ala), C1185T (Cys395Cys), G1038A (Ser346Ser), C948T (Ser316Ser), and A285G (Glu95Glu), and two missense mutations: C1502T (Ser501Phe) and G418A (Ala140Thr). These variants defined four haplotypes: H1 (1693), H2 (98), H3 (12), and a rare haplotype H4 (frequency less than five). No variants were found in the coding sequence of *SiHXK4*. In the exon region of *SiHXK5*, two synonymous mutations (G1233A (Ala411Ala) and C735T (Gly245Gly)) were identified, forming three haplotypes: H1 (1285), H2 (443), and H3 (94). *SiHXK6* showed five variant types in the exon region, including four synonymous mutations: C1320T (Gly440Gly), T1161C (Val387Val), C1131T (Pro377Pro), and T798C (Leu266Leu), and one missense mutation: G457A (Val153Ile). These formed five haplotypes: H1 (1387), H2 (235), H3 (128), H4 (37), and H5 (15).

An association study of the major haplotypes with plant height and yield-related traits was further performed using data from 961 accessions investigated in the field in multiple environments [33]. The results showed that the plant height of SiHXK3-H1 was significantly taller than SiHXK3-H2 in four environments. Additionally, the SiHXK3-H1 haplotype exhibited higher panicle length, panicle diameter, panicle weight, and panicle grain weight compared to the SiHXK3-H2 haplotype. Taken together, SiHXK3-H1 might be a superior haplotype for high yield, making it a promising target for breeding new high-yield varieties. This analysis highlights the significant genetic diversity within the HXK gene family in foxtail millet and their potential roles in agronomic traits.

## 3. Discussion

### 3.1. Identification and Characterization of HXK Genes for Foxtail Millet

Hexose sugars function both as signaling molecules and as the primary carbon source for energy, storage, and cell wall synthesis in plants. Their phosphorylation by *HXKs* converts them into metabolically active forms and integrates carbon into various metabolic pathways. Moreover, *HXKs* are central to complex signaling networks that regulate gene expression, hormone interactions, stress responses, and overall plant growth and development [4,6,34]. A comprehensive genome-wide analysis was conducted to elucidate the roles of *HXK* genes in foxtail millet, focusing on growth regulation and abiotic stress resistance. Six *HXK* genes were identified and characterized regarding their gene/protein structures, conserved motifs, evolutionary history, expression patterns, subcellular localization, and stress responses.

A high similarity in protein sequences generally indicates conserved functions. According to the well-characterized tertiary structures of *HXKs* from yeast and mammals, *HXK* proteins consist of large and small domains, primarily housing the sugar binding site/core along with four additional peptide segments (Loops 1–4) [29,35]. These structures also demonstrate that most conserved amino acid residues are located at the interface of the two domains, forming the glucose and ATP binding sites [35]. In the current study, multiple sequence alignments of the *SiHXKs* in foxtail millet revealed highly conserved regions, consistent with known *HXKs*, including motifs corresponding to phosphate 1, connect 1, phosphate 2, adenosine, and connect 2.

To investigate the role of *HXK* genes in abiotic stress, we examined their expression profiles in seedling roots under stress conditions induced by 20% PEG 6000, 200 µmol/L NaCl, and 1 µmol/L BR. With the exception of *SiHXK2*, under BR treatment, the expression levels of all six *HXK* genes were significantly upregulated at 6 and 12 h under these conditions (Figure 5). This consistent temporal upregulation suggests that the *SiHXK* gene family is likely involved in mediating plant responses to abiotic stress, thereby contributing to stress resistance. Similarly, the Prunus *HXK3* genes in Arabidopsis transgenic plants [22], the *GmHXK2* and *GmHXK15* genes in soybean [21,23], the TaHXK7-1A gene in wheat [36], and the *ZmHXK5* and *ZmHXK6* genes in maize [24] also exhibit abiotic stress resistance. These observations support the potential role of *SiHXK* genes in regulating stress-responsive pathways and provide a strong basis for further studies on their molecular mechanisms in abiotic stress resistance.

### 3.2. Evolutionary Patterns of HXK Genes in Foxtail Millet, Rice, Sorghum, and Green Foxtail Millet

A major driving force in genome evolution is provided by gene duplication events, which result in the generation of new gene copies that can acquire novel functions within gene families [37]. In this study, three species were selected for evolutionary analysis with foxtail millet. Rice, a well-established model crop, has been extensively utilized in gene cloning studies through BLAST comparisons with its homologs in foxtail millet. Furthermore, green foxtail millet is recognized as the wild progenitor of cultivated foxtail millet, and a close homologous relationship with foxtail millet is exhibited by sorghum. These species were chosen to comprehensively elucidate the evolutionary relationships of foxtail millet.

Divergence times of orthologous gene pairs were estimated (Table 2). It is suggested by our results that large-scale duplication events of *HXK* genes between foxtail millet and rice occurred approximately 37.31 to 336.15 million years ago (MYA). For foxtail millet and sorghum, it is estimated that these events occurred around 335.65 MYA, whereas for foxtail millet and green foxtail millet, they are likely to have taken place between 1.18 and 154.84 MYA. Notably, a greater number of orthologous gene pairs was observed between foxtail millet and green foxtail millet. This finding is consistent with the observations reported by Doust et al. [38], in which it was proposed that foxtail millet was domesticated from a wild ancestor closely related to green foxtail millet, thereby further reinforcing the close evolutionary relationship between them. Collectively, the evolutionary trajectories and functional diversification within this gene family are further elucidated by these insights into the duplication and divergence of *HXK* genes across these species.

### 3.3. Expression Analysis and Subcellular Localization of Foxtail Millet HXK Genes

The expression levels of genes in different tissues, along with information on their subcellular localization, provide essential insights into their biological functions. In Arabidopsis, most *HXK* genes are widely expressed across all tissues, whereas *AtHKL3* is exclusively expressed in flowers [7]. Similarly, the *OsHXKs* in rice show broad tissue expression, with *OsHXK10* being flower-specific and *OsHXK1* in any tissue [8]. The transcriptional presence of all *HXK* genes in major tissues, except *AtHKL3*, *OsHXK1*, and *OsHXK10*, suggests that each *HXK* plays a unique or redundant role in various tissues or organs. In this study, the expression levels of *HXK* genes in foxtail millet were examined during the booting, heading, flowering, and maturity stages across roots, stems, nodes, leaves, panicles, pollen, and seeds (Figure 4). The results revealed distinct expression profiles for the foxtail millet *HXK* gene family. Notably, *SiHXK3* showed minimal expression, being confined to the secondary branches, pollen, panicles, anthers, and seeds, with no expression detected in other tissues.

**Figure 6:**
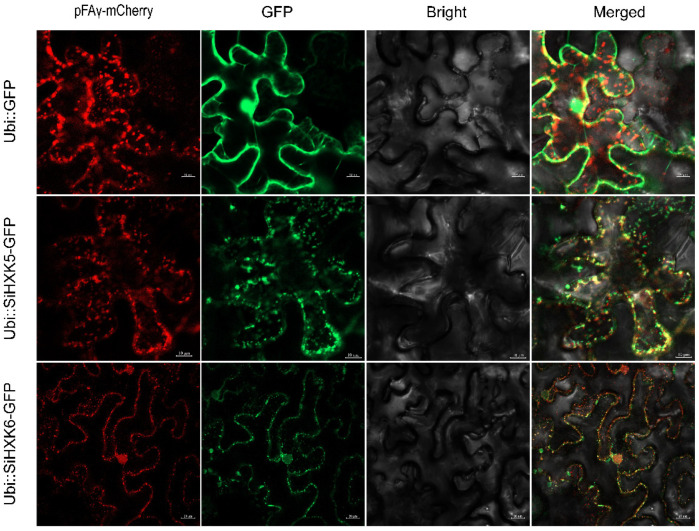
Subcellular localization of SiHXK proteins (*SiHXK5* and *SiHXK6*) in tobacco leaves. The empty vector Ubi::GFP was used as control, and the pFAy-mCherry was used as a reference for mitochondria localization. Bars for Ubi::GFP and Ubi::SiHXK5-GFP were 10 μm, and for Ubi::SiHXK6-GFP were 20 μm.

*HXKs* are classified into four subcellular localization types (A–D), with these variations contributing to the functional diversification in plants [5,6]. D-type *HXKs*, mitochondrial proteins found only in mosses, are absent in higher plants [11]. The other three types are widespread in higher plants. A-type *HXKs* possess an N-terminal chloroplast signal and typically localize to chloroplasts, and these are found in mosses and higher plants like Arabidopsis, tobacco, rice, and tomato [5,7,8,31,39]. B-type *HXKs*, the majority group in the plant *HXK* family, are characterized by highly hydrophobic helices and are associated with mitochondria; some B-type *HXKs* with nuclear localization sequences can translocate to the nucleus [8,16,40,41,42]. C-type *HXKs*, lacking signal peptides or membrane anchors, are considered to be cytoplasmic proteins, present in mosses and monocots like rice [5,8,11]. Through phylogenetic analysis, we identified four B-type *HXKs* (*SiHXK2*, *SiHXK3*, *SiHXK5*, and *SiHXK6*) and two C-type *HXKs* (*SiHXK1* and *SiHXK4*) in foxtail millet (Figure 1). Initially, we aimed to determine the cellular localization of two *HXKs* from each type, but due to the unsuccessful cloning of the C-type *HXKs*, we focused on B-type *HXKs*, specifically *SiHXK5* and *SiHXK6*, for further analysis (Figure 6). Our results demonstrated that *SiHXK5* and *SiHXK6* localized to the mitochondria in tobacco leaf cells (Figure 6), consistent with the localization of *OsHXK5* and *OsHXK6* in rice [16]. These findings provide valuable insights into the distinct roles and functional diversification of *HXK* genes in foxtail millet, contributing to our understanding of their involvement in various physiological processes and stress responses.

### 3.4. Identification of Superior HXK Haplotypes in Foxtail Millet for Breeding Applications

**Figure 7:**
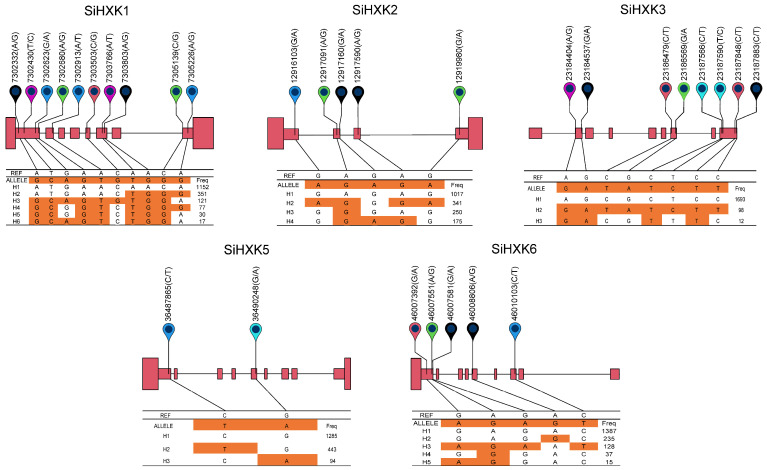
Haplotype analysis of *SiHXKs* in 1844 *setaria* accessions. The colored markers indicate SNP positions and their variant types. Red rectangles represent exons, with the leftmost rectangle indicating the 3′ UTR and the rightmost rectangle indicating the 5′ UTR, straight lines represent introns. The REF column shows the reference sequence bases, while the ALLELE column displays the variant bases for different haplotypes. The Freq column indicates the frequency of each haplotype. The orange in the table highlights bases in haplotypes that differ from the reference sequence.

**Figure 8:**
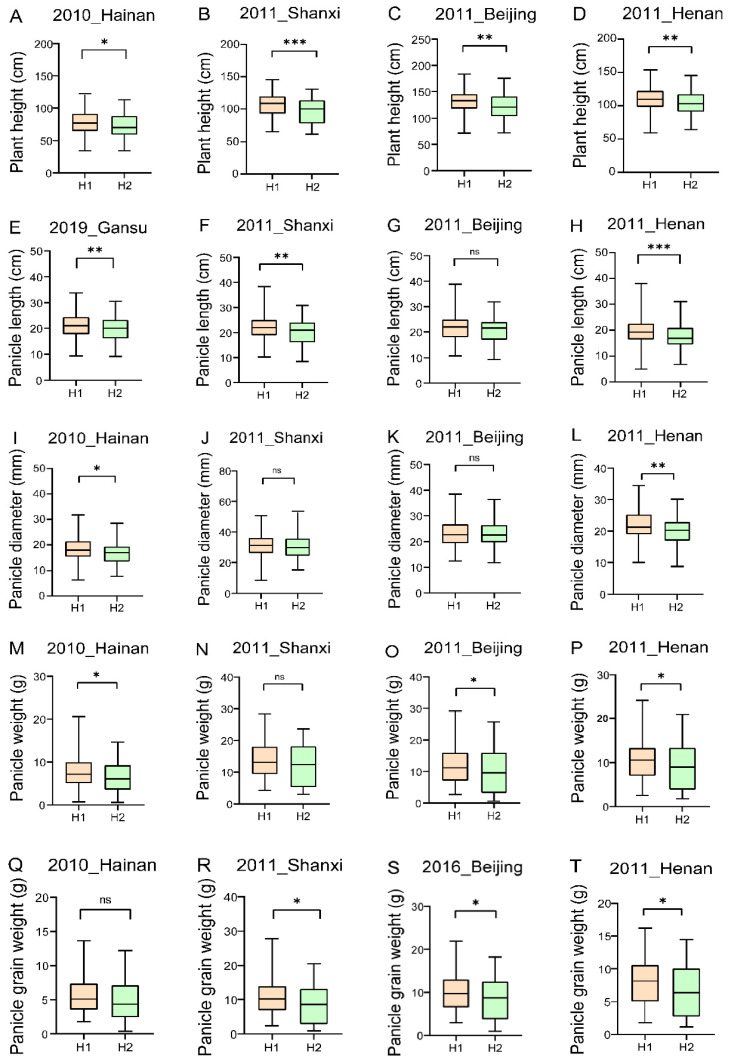
Association analysis of *SiHXK3* haplotypes with agronomic traits in setaria germplasm. Statistical significance was determined using Student’s *t* tests: ns, not significant; *, *p* < 0.05; **, *p* < 0.01; ***, *p* < 0.001.

Haplotype analysis of the *HXK* genes family in foxtail millet revealed that while the conserved exon coding region of *SiHXK4* had no detected variations, *SiHXK2* and *SiHXK5* exhibited synonymous mutations, and *SiHXK1*, *SiHXK3*, and *SiHXK6* harbored missense mutations (Figure 7). In particular, two missense mutations (C1502T [Ser501Phe] and G418A [Ala140Thr]) in *SiHXK3* were associated with significantly greater plant height and superior yield-related traits in the H1 haplotype compared to the H2 haplotype, identifying H1 as a superior haplotype for breeding (Figure 8).

Similarly, in rice, it was found that the loss of function of *OsHXK3*—a mitochondria-associated, non-catalytic hexokinase-like protein involved in regulating gibberellin biosynthesis and spikelet husk cell expansion—results in reduced grain size, whereas the overexpression of *OsHXK3* increases yield [43]. These findings collectively underscore the crucial role of *HXK* genes in regulating growth and yield in cereal crops and highlight their potential as targets for molecular breeding.

## 4. Materials and Methods

### 4.1. Identification of HXKs in Foxtail Millet

Foxtail millet (version 2.2), green foxtail (version 2.1), and sorghum genome (version 3.1.1) data were retrieved from the Phytozome database (https://phytozome-next.jgi.doe.gov/, accessed on 1 November 2024). Meanwhile, rice genome data were obtained from the Rice Data Center (https://www.ricedata.cn/, accessed on 1 November 2024), and Arabidopsis genome data were downloaded from the TAIR database (https://www.arabidopsis.org/, accessed on 1 November 2024).

The HMM (PF03055) for the conserved domains of *HXK* proteins was obtained from the Pfam database (version 37.2, http://pfam.xfam.org/). Using the downloaded HMM model, HMMER (version 3.4) software was employed on a Linux server to identify *HXK* genes in the genomes of foxtail millet. The specific parameters used were as follows: hmmsearch --cut_tc --domtblout. The results were further filtered with an E-value < 0.001. The awk command (awk ′{print $1}′) on the Linux server was used to extract a list of all *HXKs* genes. Simultaneously, BLASTp was performed on a Linux server to compare the protein sequences of six *HXKs* from Arabidopsis with the entire proteome of foxtail millet, using an evalue threshold of 1 × 10^−30^. The awk command (awk ‘{print $1}’) was utilized on the Linux server to extract a comprehensive list of all identified *HXK* genes. The intersection of the *HXK* gene lists obtained through the two approaches was taken as the final set of candate *HXK* genes.

Extract protein and CDS sequences were obtained using the HXK gene IDs from rice [8], Arabidopsis [7], sorghum [26], and green foxtail millet [44]. The R package Peptides (version 2.4.6) [45] was then used to calculate the molecular weight (MW) and isoelectric point (PI).

### 4.2. Gene Structure, Conserved Domains, and Phylogenetic Analysis

Multiple sequence alignments and visualizations were performed using the R package msa [28]. The alignment results were used to construct a phylogenetic tree on the Linux server with IQ-TREE (version 2.3.6) [46] using the maximum likelihood method, with parameters: -m MFP -bb 1000. The phylogenetic tree was visualized using the online tool iTOL (https://itol.embl.de, accessed 20 December 2024). Conserved motif analysis of HXK protein sequences was conducted on a Linux server using MEME software (version 5.5.5) with the following parameters: up to 10 motifs were detected, with a minimum motif width of 6 amino acids and a maximum motif width of 200 amino acids [47].

The foxtail millet genome annotation file (version 2.2) and GSDS (https://gsds.gao-lab.org/) software [48] were employed for the gene structure visualization of *HXK* genes in foxtail millet.

### 4.3. Ka/Ks Analysis of Orthologous Genes Between Foxtail Millet and Arabidopsis, Rice, Sorghum, and Green Foxtail Millet

Synonymous substitution rate (Ks) and non-synonymous substitution rate (Ka) were calculated using KaKs_Calculator [49], with genetic code Table 1 (standard code) and the YN method. Evolutionary divergence times were calculated using the formula T = Ks/2λ (λ = 6.5 × 10^−9^) [50]. Ka/Ks > 1 indicates positive selection, Ka/Ks = 1 indicates neutral selection, and Ka/Ks < 1 indicates negative or stabilizing selection [51].

### 4.4. Cis-Acting Element Analysis of Foxtail Millet HXK Genes

The 2000 bp upstream sequences of the foxtail millet *HXK* genes were extracted as promoter sequences and submitted to the PlantCARE database (http://bioinformatics.psb.ugent.be/webtools/plantcare/html/, accessed 21 December 2024) [52] for cis-acting element analysis.

### 4.5. Tissue-Specific Expression Analysis of Foxtail Millet HXK Genes

Expression data for *HXK* genes in roots, stems, leaves, nodes, panicles, pollen, and seeds at different growth stages (booting, shooting, flowering, and mature) of Yugu1 were obtained from the Setaria-db database (http://111.203.21.71:8000, accessed 8 November 2024) [32]. Expression heatmaps were constructed based on the differential expression of each gene across various organs and developmental stages, and visualized using the R package pheatmap (version 1.0.12) [53].

### 4.6. Abiotic Stress Response of Foxtail Millet HXKs Genes

In this experiment, Yugu1 was used as the material. Seeds were germinated on moist filter paper in petri dishes at 28 °C under a 14 h light/10 h dark cycle. Once the seedlings reached 2 cm in height, they were transferred to hydroponic boxes containing 1/2 Hoagland nutrient solution. After two weeks, abiotic stress treatments, including 20% PEG 6000, 200 μmol/L NaCl, and 1 μmol/L BR, were applied. Root samples were collected at 0 h, 1 h, 6 h, 12 h, and 24 h post-treatment, rapidly frozen in liquid nitrogen, and stored at −80 °C. A concentration of 200 μmol/L NaCl was chosen to simulate salt stress conditions typical of normal agricultural soils, where salt concentrations generally range from several hundred to a few thousand μmol/L NaCl. This concentration is lower than those commonly used in foxtail millet salt stress studies (e.g., 0.15 mol/L, 0.13 mol/L, 0.17 mol/L, and 0.22 mol/L NaCl) [54,55,56]. However, considering the moderate salt sensitivity of foxtail millet, as described in the Chinese national standard for saline–alkali soil classification, the choice of 200 μmol/L NaCl was intended to better reflect salt stress conditions resembling those in normal agricultural soils.

Total RNA was extracted from the treated samples, and its integrity was assessed using 1% agarose gel electrophoresis, with clear and bright 28S and 18S bands indicating intact RNA. RNA concentration was measured using a Nanodrop ND 1000 spectrophotometer (NanoDrop, Wilmington, DE, USA). The extracted RNA was reverse transcribed into cDNA using the PrimeScriptTM II 1st Strand cDNA Synthesis Kit (Takara, Osaka, Japan, Cat No. 6210A). Quantitative real-time PCR (qRT-PCR) was performed using designed primers and Cullin reference gene primers (Table S3) with the Realtime PCR Supermix SYBR green with anti-Taq kit(Aikori Biotech, Changsha, China). The expression data were analyzed using the 2^−ΔΔCT^ method. Each experiment was conducted with three technical replicates.

### 4.7. Subcellular Localization of Foxtail Millet HXK Proteins

The full-length CDS regions of *SiHXK5* and *SiHXK6* genes was integrated into the vector containing the green fluorescent protein (GFP) sequence, driven by the ubiquitin (Ubi) promoter. The full-length CDS regions of *SiHXK5* and *SiHXK6* genes was amplified by PCR using primers (Table S3). The GFP sequence was fused to the C-terminus of the SiHXK proteins to ensure proper localization without interfering with their native function. The constructs Ubi::SiHXK5-GFP and Ubi::SiHXK6-GFP were introduced into Nicotiana benthamiana leaves using Agrobacterium tumefaciens strain GV3101, with Ubi::GFP (empty vector) as a negative control. After three independent transformation experiments, the infiltrated leaves were incubated for three days, and fluorescence signals were examined using a confocal laser scanning microscope. Fluorescence colocalization was assessed in multiple fields of view to confirm consistent mitochondrial localization.

### 4.8. Haplotype Variation Analysis of Foxtail Millet HXK Genes

Haplotype analysis of six *HXK* genes was conducted using sequencing data from 961 core foxtail millet germplasm materials [33]. Agronomic traits such as plant height, panicle length, panicle diameter, panicle weight, and panicle grain weight were surveyed in Hainan (2010), Henan (2011), Beijing (2011), Shanxi (2011), and Gansu (2019). The association between locus variations and phenotypes was analyzed, and the significance of differences was assessed using T-tests in GraphPad Prism (version 8.0.2) [57] for visualization.

## 5. Conclusions

Six *HXK* genes in foxtail millet were first identified and subsequently grouped into three distinct subgroups. All *HXKs* exhibited diverse expression patterns. Notably, our findings revealed that *SiHXK5* and *SiHXK6* are localized to the mitochondria in tobacco leaf cells, aligning with the mitochondrial localization of *OsHXK5* and *OsHXK6* in rice. The haplotype analysis of *SiHXK3* genes revealed that the H1 haplotype is associated with increased plant height and grain yield, indicating its potential for favorable selection in breeding programs.

## Figures and Tables

**Table 1:** Sequence characteristics of the genes of *HXK* families in *Setaria italica*.

Species	Gene ID	Chromosome Location	CDS Length (bp)	Protein		
				Length (aa)	MW (kDa)	pI
*Setaria italica*	*SiHXK1*	Chr3:7302120.7305664	1400	461	50.05213	5.29
	*SiHXK2*	Chr3:12915471.12920520	1543	508	54.85187	6.39
	*SiHXK3*	Chr3:23183401.23187890	1527	503	54.35627	5.17
	*SiHXK4*	Chr5:11737238.11741690	1424	469	51.01015	5.03
	*SiHXK5*	Chr5:36487149.36492845	1536	506	54.81674	6.40
	*SiHXK6*	Chr5:46006911.46013308	1512	498	53.39601	6.04

CDS, coding sequence; MW, molecular weight of protein; pI, protein isoelectric point.

**Table 2:** Divergence between orthologous gene pairs.

Orthologous Gene Pairs		Ka	Ks	Ka/Ks	MYA
*SiHXK1*	*SvHXK1*	0.0038	0.0154	0.2485	1.18
*SiHXK1*	*SvHXK4*	2.1465	2.0996	1.0223	161.15
*SiHXK1*	*OsHXK1*	0.1782	4.1311	0.0431	317.78
*SiHXK2*	*SvHXK5*	2.7857	1.1205	2.4862	86.19
*SiHXK2*	*OsHXK10*	0.2456	1.7110	0.1436	131.62
*SiHXK3*	*SvHXK6*	5.1355	1.8350	2.7986	141.15
*SiHXK3*	*SvHXK3*	5.1259	1.9357	2.6482	148.90
*SiHXK5*	*SvHXK2*	2.8365	2.0129	1.4092	154.84
*SiHXK5*	*OsHXK6*	0.0413	0.4850	0.0851	37.31
*SiHXK5*	*OsHXK10*	0.2202	3.1476	0.0699	242.12
*SiHXK5*	*SbHXK2*	0.2723	4.3634	0.0624	335.65
*SiHXK6*	*SvHXK6*	5.1138	1.3859	3.6898	106.61
*SiHXK6*	*SvHXK3*	5.1106	1.7442	2.9301	134.17
*SiHXK6*	*SvHXK4*	5.1728	1.0347	4.9992	161.51
*SiHXK6*	*SvHXK1*	5.1835	1.2253	4.2304	94.25
*SiHXK6*	*OsHXK1*	0.4022	4.3699	0.0920	336.15

Ka: non-synonymous substitution rate; Ks: synonymous substitution rate; MYA: million years ago.

## Data Availability

The data for this study are available from the internet links shown in the paper.

## Supplementary Materials

The following supporting information can be downloaded at the following site: https://www.mdpi.com/article/10.3390/ijms26051962/s1.
