# Supplementary material for: Genomic Analysis of Hexokinase Genes in Foxtail Millet (Setaria italica): Haplotypes and Expression Patterns Under Abiotic Stresses
Source: Int J Mol Sci. 2025 Feb 24;26(5):1962. doi: 10.3390/ijms26051962 (PMC11900577; doi:10.3390/ijms26051962)
Supplement: Supplementary file 1 [file ijms-26-01962-s001.zip › Figure S1 Heatmap of cis-acting elements.pdf]

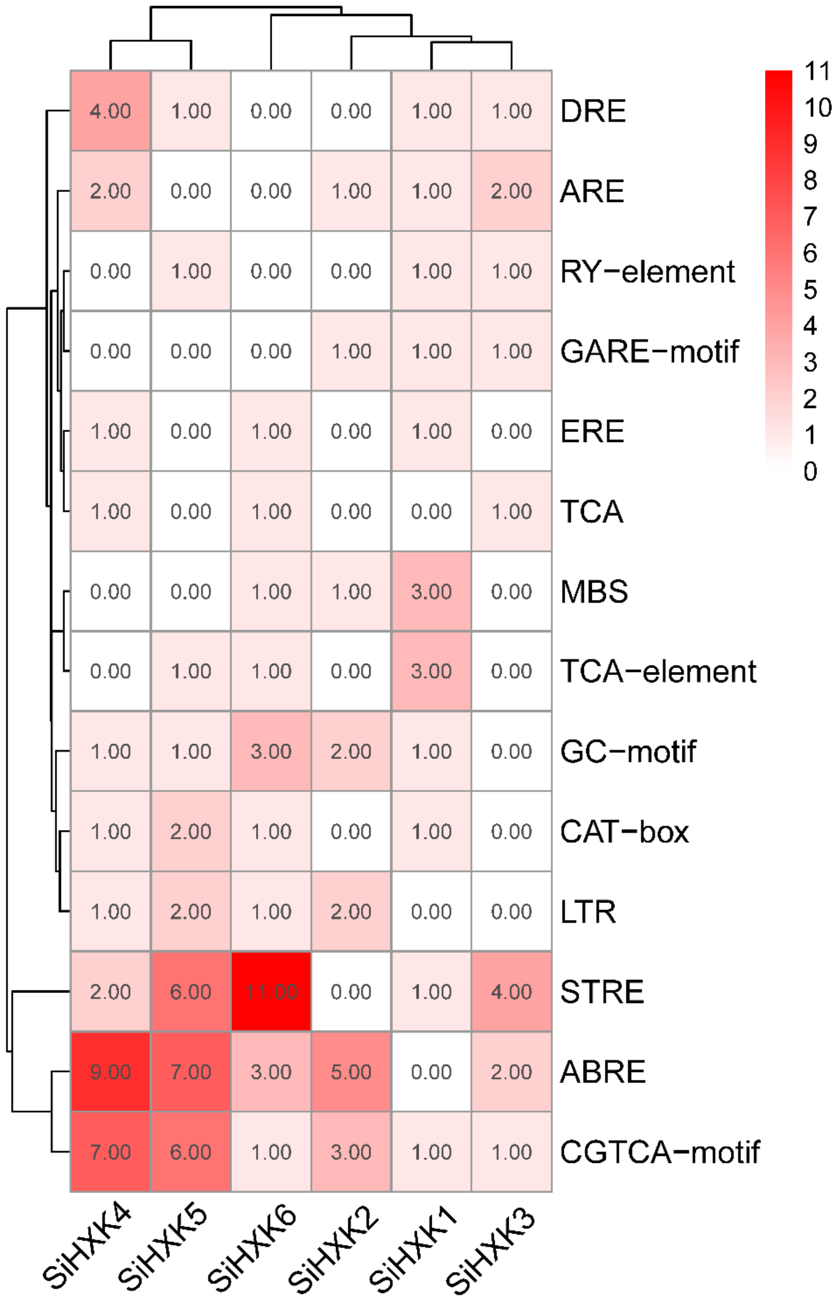

Figure S1. Heatmap of cis-acting elements. The numbers in the figure indicate the number of elements, with the element names shown on the right.
