# Supplementary material for: Genomic Analysis of Hexokinase Genes in Foxtail Millet (Setaria italica): Haplotypes and Expression Patterns Under Abiotic Stresses
Source: Int J Mol Sci. 2025 Feb 24;26(5):1962. doi: 10.3390/ijms26051962 (PMC11900577; doi:10.3390/ijms26051962)
Supplement: Supplementary file 1 [file ijms-26-01962-s001.zip › Table S1 Protein sequences.pdf]

Table S1. Protein sequences.

>*SiHXK1*
MVAAAAERVVAELREACAAPAARLNEVAAAMEAEMRAGLREEGGSKIKMIISYVDNLPT
GNEEGFFYSLDLGGTNFRVLRVQLAGKEKRVAKRESKEVSIPPHLMSGNASELFGFIASAL
AKVIADEGRNDVFEDKQRELGFTFSFPVRQTSIASGTLIKWTKAFSIDDAVGEDVVAELRT
AMEKHGVHMRVAALINDTVGTLAAGRYNDEDVVIGVILGTGSNAAYVEEASAIPKFEGEL
PKSGNMVINTEWGNFYSSCLPITEYDQALDEESLNPGEQIFEKLISGMYLGEIVRRVLLKIA
SQSTLFGKVNHTKLKTRFILRTPDISAMHHDETPDLRIVAEKLADNFKIKDTSLETRKMVV
EICDVVTSRSARLAAAGIVGILRKIGRAVPGDERRSVVAIDGGLFEHYAEFRQCLESTLVEL
LGEEASRSVAVKLTKDGSGLGAALIAAAHSQYQH*
>*SiHXK2*
MGKAAAVGTAVVVCAAVGVAVVLARRRRRDAELLGSADADRKRRAAAVIEEVERSLAT
PTALLRSIADAMVTEMERGLRADIHAQLKMLISYVDNLPTGDEHGLFYALDLGGTNFRVL
RVQLGGREKRVVKQQYEEVSIPPHLMVGTSLELFDFIAAALAKFVDTEGEDFHLPEGRQR
ELGFTFSFPVNQTSISSGTLIKWTKGFSVNGMVGEDVVSELSKAMERQGLDMKVTALVND
TVGTLAGGRYMDNDVVAAVILGTGTNAAYVEHANAIPKWTGLLPKSGNMVINTEWGSF
KSDKLPLSEYDKAMDFESLNPGEQIYEKLISGMYLGEIVRRILLKLAHDASLFGDVVPSKL
EQPFVLRTPDMSAMHHDSSHDLKILGAKLKDIVGVADTSLEVRYITRHICDLVAERGARLA
AAGIYSILKKIGRDKVPSNGSKMPRTVIALDGGLYEHYKKFSSCVETTLTDLLGEEASSSV
VAKLANDGSGIGAALLAASHSQYAEAD*
>*SiHXK3*
MVIHMCMRWSRSEEGIYYAIDLGGTSFRVMKLELGPGSMVINKKVEHQPIPEELTKGTSED
LFNLIASALKNFIEREGGKDEGRALGFTFSFPVRQISISSGSLIRWTKEFSIEEAVGMDVAQC
LNEALVRNGLNLQVTALVNNAVGTLAMGHYYDEDTVAAVIIGAGTNASYIERTATITKCQ
ALLTNSDITVVNVEWGSFRPPQIPLTPYDICFNVEKDRNHYDQAFEKMISGVYLGEIARLV
LQGMAQESDVFGSSVDFLSTPFIFSTPCLAAIREDDSPDLRVVGRVLEEQLKIQDVPLKTRR
LVVRICDIVTRRAARLAAAGIVAVLQKIGRDGTLCGTSMVRKIRGKPKRSVVAIEGGLYQG
YSVFREYLNEAVDEILGDEIASTVSLRVMEEGSGIGAALLAASYSSTRQNSA*
>*SiHXK4*
MAAAEAVAMAEQVVADLREKCETPPELLREVASAMAHEMGAGLEKDGGSRVNMLLSYV
DKLPTGREEGLFYGLDLGGTNFRVLRVQLGGIEKHVVNRESREVSIPPQLMSGSSSELFGFI
ASELAKFVADEEKCANVLSNGKKRELGFTFSFPVKQRSVASGTLVKWTKAFSIDDAVGED
VVAELQTAMEKQGLDMHVAALINDAVGTLAGARYYDRDVVAGVIFGTGTNAAYVEKAN
SIPKWEGELPNSGDMVINMEWGNFCSSHLPVTEYDRELDKESLNPGEQIYEKLMSGMYL
GEIVRRVLLKMSLQSSIFGNIDHTKLKTHFLLRTPHISAMHHDETPDLKIVAEKLEENLEITG
TSLETRKLVVEICDIVARRAARLAAAGLAGILKKLGRDGNAQEQRSVIAIDGGLFEHYTKF
RECLEITLGELLAEYASKSVAVKHADDGSGIGAALIAASQSQYRNVE*
>*SiHXK5*
MGKGAVVGTAVVVCAAAAAAVGVAVVVSRRRRRREAENERKRKAAAVIEEVEQAFSTP
TALLRGIADAMVVEMERGLRADPHAPLKMLISYVDNLPTGDEHGLFYALDLGGTNFRVIR
VQLGGREKRVVKQQYEEVSIPPHLMVGTSTELFDFIAAELEKFVRTEGEDFHLPDGKQRE
LGFTFSFPVHQTSISSGTLIKWTKGFSINGTVGEDVVAELSRAMERQGLDMKVTALVNDTV
GTLAGGRYVDNDVAAAVILGTGTNAAYVEHANAIPKWNGLLPRSGNMVINMEWGNFRS
DKLPSSEYDKALDFESLNPGEQIYEKMISGMYLGEIVRRILLKLAHDASLFGDVVPPKLEQ

LFVLRTPDMSAMHHDTSHDLKHLGAKLKDILGVPDTSLEARYITLHVCDLVAERGARLAA
AGIYGILKKLGKDKLPNDFSQQRTVVAMDGGLYEHYKKFSACLEATLTDLLGEEAASSVV
VKLANDGSGIGAALLAASHSQYAEAA*

>*SiHXK6*

MVVEMHAGLASDGGSKLKMLLTFVDALPTGNEEGIYYAIDLGGTNFRALRVEVGSGSVV
TSRKVELPIPEELTKGTIEELFNFVAIALKDFVEREDGKDEKRALGFTFSFPVRQTSVSSGSL
IRWTKGFSIENAQVGKDVAQCLNEALAMSGLNVRVTALVNDTVGTLALGHYHDEDTVAA
VIIGAGTNACYIERTDAIIKCQGLLTNSGGMVVNMEWGNFWSSHLPRTPYDISLDDETQNR
NDQGFEKMISGIYLGEIARLVLHRMALESDVFGDAADNLSTPFTLSTPLLAAIREDDSPDLS
EVRRILQEHLKIPDTPLKTRRLVVKVCDIVTRRAARLAAAGIVGILKKLGRDGSGVASSGR
TRGQPRRSVVAIEGGLYQGYPVFREYLDEALVEILGEEVARTVALRVTEDGSGVGAALLAA
VYSSNRQQGSI*

>*OsHXK1*

MAAAAVAADQKVVTMTSLREGCACAAPPAAAAPPMPKMAAAQRVVAELREACATPAAR
LAEVAAAMAGEMEAGLAVEGGSSEMKMIVSYVDSLPTGGEEGSYYALDLGGTNFRVLRV
RLAGGGVAERVAREVPIPPGLMSGGGATSECLFGFIASALAEFVGEEEEEGGLDGGERELG
FTFSFPVHQTSIASGTLIRWTKAFAVDDAIGEDVVAALQAAMSERGLDMRVSALINDTVGT
LAAGSYYDEDVVAAVILGTGTNAAYVEDATAIAKLHPSQLPASNTMVINTEWGSFASPCLP
LTEFDEALDQESLNPGEQTYEKLISGMYLGEIVRRVLLKISSRCPSLLGGAGELATPFVLRT
PDVSAMHHDETPDLSIVGEKLERTLGIRGTSPEARRMVVEVCDIVATRAARLAAAGIVGIL
KKIGRVDGGEGRRRRSVVAVDGGLFEHYGKFRRCMESAVRELLGEAAAERVVVKLASDG
SGLGAALVAAAHSQRA

>*OsHXK2*

MGKAAAVGTAVVVAAAVGVAVVLARRRRRRDLELVEGAAAERKRKVAAVIEDVEHALST
PTALLRGISDAMVTEMERGLRGDSHAMVKMLITYVDNLPTGNEQGLFYALDLGGTNFRV
LRVQLGGKEKRVVQQQYEEVSIPPHLMVGTSMELFDFIASALSKFVDTEGDDFHLPEGRQ
RELGFTFSFPVSQTSISSGTLIKWTKGFSINDAVGEDVVSELGKAMERQGLDMKIAALVND
TVGTLAGGRYADNSVVAAIILGTGTNAAYVENANAIPKWTGLLPRSGNMVINTEWGSFKS
DKLPLSEFDKAMDFESLNPGEQIYEKLISGMYLGEIVRRILLKLAHDAALFGDVVPSKLEQ
PFVLRTPDMSAMHHDSSHDLKTVGAKLKDIVGVPDTSLEVRYITSHICDIVAERAARLAA
AGIYGVLKKLGRDKMPKDGSKMPRTVIALDGGLYEHYKKFSSCLESTLTDLLGDDVSSSV
VTKLANDGSGIGAALLAASHSQYAEID

>*OsHXK3*

NFVALALKNFLEGEDDQDGKMALGFTFSFPVRQISVSSGSLIRWTKGFSIRDTVGRDVAQC
LNEALANCGLNVRVTALVNDTVGTLALGHYYDEDTVAAVIIGSGTNACYIERTDAIIKCQG
LLTNSGGMVVNMEWGNFWSSHLPRTPYDILLDDETHNRNDQGFEKMISGMYLGEIARLV
FHRMAQESDVFGDAADSLSNPFILSTPFLAAIREDDSPDLSEVRRILREHLKIPDAPLKTRR
LVVKVCDIVTRRAARLAAAGIVGILKKLGRDGSGAASSGRGRGQPRRTVVAIEGGLYQGY
PVFREYLDEALVEILGEEVARNVTLRVTEDGSGVGAALLAAVHSSNRQQQGGPI

>*OsHXK4*

NTEWGAFSDGLPLTEFDREMDDESINPGEQIFEKTISGMYLGEIVRRVLVKMAEVSDLFGH
SFPKKLAEPFVLRTPHLCAMQQDTSDNLGEVESILSDVIGVSQASLLARRVTVEVSDCIIRR
GGRLAGAGIVGILEKMENDSRGHIFGRRTVVAMDGGLYEKYPQYRRYMKEAVAELLGPE
RSNRIAIEHTKDGSGIGAALLAAANSKYAAAQISTR

>*OsHXK5*

MEGRAAGWVRVAAVGWAVAACAVAAGMVARRGAARVRWNRAVAVVRDLEERCATPAEL
LQRVVNSLAIEMFAGLASDGGSKVRMLLTCVDALPDGSEEGISYAIDLGGTSFRVLKVELG
AGSTIINRKVEHQPIPENLTKGTSDDLFNFIASALKNFIEREGGEVEGRALGFTFSFPVRQTS
ISSGTLIRWTKEFSIEEAVGKDVAQCLNEALARNGLNMKVNVLVNNTVGTLALGHYYDD
DTVAAVIIGAGTNACYIERNDAIIKSLGRVTNSERTVVNVEWGSFRPPQIELTPYDICFNNET
WNYYDQGFEKMISGVYLGEIARLVFQKMAEESDIFGTAVDGLSTPFVLSTPNLAAIREDDS
PDLREVGKILEEHLKLPDVPLKTRKLVARVSDIITRRAARLAAAIVAILQKIGCDGTLCGS
TQVRTMRGVRRRTVVAIEGGLFEGYSVFREYLNEALVEILGEEIAATVSLRVMEEGSGTGA
ALLAAAYSSARQKNSE

>*OsHXK6*

MGKGTVVGTAVVVCAAAAAAVGVAVVVSRRRRSKREAEEERRRRAAAVIEEVEQRFSTP
TALLRGIADAMVEEMERGLRADPHAPLKMLISYVDNLPTGDEHGLFYALDLGGTNFRVIR
VQLGGREKRVVSQQYEEVAIPPHLMVGTSMELFDFIAAELESFVKTEGEDFHLPEGRQREL
GFTFSFPVHQTSISSGTLIKWTKGFSINGTVGEDVVAELSRAMERQGLDMKVTALVNDTV
GTLAGGRYVDNDVAAAVILGTGTNAAYVEHANAIPKWTGLLPRSGNMVINMEWGNFKS
ERLPRSDYDNALDFESLNPGEQIYEKMISGMYLGEIVRRILLKLAHDASLFGDVVPTKLEQ
RFILRTPDMSAMHHDTSHDLKHLGAKLKDILGVADTSLEARYITLHVCDLVAERGARLAA
AGIYGILKKLGRDRVPSDGSQKQRTVIALDGGLYEHYKKFRTCLEATLADLLGEEAASSVV
VKLANDGSGIGAALLAASHSQYASVE

>*OsHXK7*

MVAAAVAAAEQVVAALREECATPAARLDGVAAAMAGEMAAGLAEEGGSKIKMIVSYVD
NLPNGTEEGLFYALDLGGTNFRVLRVQLAGKEKRVVKRESREVSIPPHLMSGNSSELFGFI
ASALAKFVADEGHNAVFNDRQRELGFTFSFPVRQTSIASGTLIKWTKAFSIDDAVGEDVVA
ELQMAMEKQGLDMRVSALINDTVGTLAAGSYYDEDIVVGVILGTGSNAAYLEKANAIPK
LEGELPKSGNMVINTEWGNFSSSCLPITEYDEALDKESLNPGEQIFEKLISGMYLGEIVRRV
LLKISLQSSIFGNLDQTKLKTRFILRTPDISVMHHDGTPDLRIVAEKLADNLKITDTSLETRK
MVVEICDIVTRRSARLAAAGIVGILRKIGRGVPGDKRKSVIAIDGGLYEHYTEFRQCLETTL
TELLGEEASKSVAVKLANDGSGLGAALIAAAHSQYLN

>*OsHXK8*

MAAVEAEKVVAELRERCATPASLLRDVAAAMAGEMGAGLEKEGGSRVKMLLSYVDKLP
TGREDGLFYGLDLGGTNFRVLKVHLGGSKKHVVNSESREVSIPPHLMSGTSSELFGFIAGE
LGKFVAEEEEGTDMPNGKKKELGFTFSFPVRQRSVASGTLVKWTKAFSIDDAVGEDVVAE
LQTAMVKQGLDMHVAALINDAVGTLAGARYYDEDVVAGVIFGTGTNAAYVEKANAIPK
WEGELPNSGDMVINMEWGNFYSSHLPVTEYDEALDKESLNPGEQIYEKLTSGMYLGEIV
RRVLLKLSLQSGIFGSIDNSKLKTCFHLRTPHISAMHHDETPDLKIVAEKLHQILEITHTSLEI
RKMVVEICDIVARRAARLAAAGVAGILMKLGRNGGINNQRSVIAIDGGLFEHYTKFRECL
ESTLGELLGEEASKSVAVKHANDGSGIGAALIAASQSR

>*OsHXK9*

MRKAAALASAAMAAAAVAVVSTVLHQRQRRAAKRSERAEAVLLRDLQERCAAPVELLR
QVADAMAAEMRAGLAAEGGSDLQMLVTYVDSLPSGGEKGMFYALDLGGTNFRVLRVQL
GGKERRIIKQDSEGISIPQHLMSSSSHELFDFVAVALAKFVASEGEDCHLPEGTQRELGFTFS
FPVKQKSLASGTLIKWTKSFAIDEMVGKDVVAELNMAIRSQGLDMKVTALVNDTVGTLA
AGRYVNHDTIAAVILGTGSNAAYIDHADAIPKWHGSLPKSGNMVCFFSPYFCSSVC

>*OsHXK10*
MHCDRSPDLRTVGAKLKDILGVQNTSLKTRRLVVDVCDIVAKRAAHLAAAGIHGILKKLG
RDVPNTDKQRTVIAVDGGLYEHYTIFAECVESTLRDMLGEDVSSTIVIKLAKDGSGIGAAL
LAAAHSQYREAEEL
>*AtHXK1*
MGKVAVGATVVCTAAVCAVAVLVVRRRMQSSGKWGRVLAILKAFEEDCATPISKLRQVAD
AMTVEMHAGLASDGGSKLKMLISYVDNLPSGDEKGLFYALDLGGTNFRVMRVLLGGKQ
ERVVKQEFEEVSIPPHLMTGGSDELFNFIAEALAKFVATECEDFHLPEGRQRELGFTFSFPV
KQTSLSSGSLIKWTKGFSIEEAVGQDVVGALNKALERVGLDMRIAALVNDTVGTLAGGRY
YNPDVVAAVILGTGTNAAYVERATAIPKWHGLLPKSGEMVINMEWGNFRSSHLPLTEFDH
TLDFESLNPGEQILEKIISGMYLGEILRRVLLKMAEDAAFFGDTVPSKLRIPFIIRTPHMSAM
HNDTSPDLKIVGSKIKDILEVPTTSLKMRKVVISLCNIIATRGARLSAAGIYGILKKLGRDTT
KDEEVQKSVIAMDGGLFEHYTQFSECMESSLKELLGDEASGSVEVTHSNDGSGIGAALLA
ASHSLYLEDS*
>*AtHXK2*
MGKVAVATTVVCSVAVCAAAALIVRRRMKSAGKWARVIEILKAFEEDCATPIAKLRQVAD
AMTVEMHAGLASEGGSKLKMLISYVDNLPSGDETGFFYALDLGGTNFRVMRVLLGGKH
DRVVKREFKEESIPPHLMTGKSHELFDFIVDVLAKFVATEGEDFHLPPGRQRELGFTFSFPV
KQLSLSSGTLINWTKGFSIDDTVDKDVVGELVKAMERVGLDMLVAALVNDTIGTLAGGR
YTNPDVVVAVILGTGTNAAYVERAHAIPKWHGLLPKSGEMVINMEWGNFRSSHLPLTEY
DHSLDVDSLNPGEQILEKIISGMYLGEILRRVLLKMAEEAAFFGDIVPPKLKIPFIIRTPNMS
AMHSDTSPDLKVVGSKLKDILEVQTSSLKMRKVVISLCNIIASRGARLSAAGIYGILKKIGR
DATKDGEAQKSVIAMDGGLFEHYTQFSESMKSSLKELLGDEVSESVEVILSNDGSGVGAA
LLAASHSQYLELEDDSETS*
>*AtHXK3*
MSLMFSSPVVTPALGSFTFSSRPRSNYIVMSAVRSNSASTCPILTKFQKDCATPTPYLRNVA
NAIADDMRDGLAVEGGGDLEMILTFVDALPSGNEEGLFYALDLGGTNFRVRSVQLGGKK
ERVLATESEQISISQKLMIGTSEELFGFIASKLANFVAKEKPGRFLLEEGRKRELGFTFSFPV
KQTSIDSGTLSKWTKGFKVSGMEGKNVVACLNEAMEAHGLDMRVSALVNDGVGTLAGA
RYWDEDVMVGVILGTGTNACYVEQKHAIPKLRSKSSSGTTIINTEWGGFSKILPQTIFDLE
MDETSLNPGEHLYEKMISGMYLGEIVRRVLLHMCETSDLFGHFAPAKLSTPLALRTEHLCK
MQEDNTDDLRDVGSILYDFLDVEANMNARRRVVEVCDTVVKRGGRLAGAGIVAILEKIE
KDTKRMGSGKRTVVAMDGALYEKYPQYRQYMQDALVELLGHKLASHVAIKHTKDVSGL
GAALLAATNSIY*
>*AtHKL1*
MGKVAVAFAAVAVVAACSVAAVMVGRRMKSRRKWRTVVEILKELEDDCDTPVGRLRQV
VDAMAVEMHAGLASEGGSKLKMLLTFVDDLPTGREKGTYYALHLGGTYFRILRVLLGDQ
RSYLDVQDVERHPIPSHLMNSTSEVLFNFLAFSLERFIEKEENGSDSQGVRRELAFTFSFPV
KHTSISSGVLIKWTKGFEISEMVGQDIAECLQGALNRRGLDMHVAALVNDTVGALSLGYY
HDPDTVVAVVFGTGSNACYLERTDAIIKCQGLLTTSGSMVVNMEWGNFWSSHLPRTSYDI
DLDAESSNANDMGFEKMISGMYLGDIVRRVILRMSEDSDIFGPISPVLSEPYVLRTNSVSAI
HEDDTPELQEVARILKDIGVSDVPLKVRKLVVKICDVVTRRAGRLAAAGIAGILKKIGRDG
SGGITSGRSRSEIQMQKRTVVAVEGGLYMNYTMFREYMEEALVEILGEEVSQYVVVKAM
EDGSSIGSALLVASLQS*

>*AtHKL2*
MGKVLVMLTAAAAVVACSVATVMVRRRMKGRRKWRRVVGLLKDLEEACETPLGRLRQ
MVDAIAVEMQAGLVSEGGSKLKMLLTFVDDLPNGSETGTYYALHLGGSYFRIIKVHLGGQ
RSSLEVQDVERHSIPTSLMNSTSEVLFDFLASSLQRFIEKEGNDFSLSQPLKRELAFTFSFPV
KQTSISSGVLIKWTKGFAISEMAGEDIAECLQGALNKRGLDIRVAALVNDTVGALSFGHFH
DPDTIAAVVFGTGSNACYLERTDAIIKCQNPRTTSGSMVVNMEWGNFWSSRLPRTSYDLE
LDAESMNSNDMGFEKMIGGMYLGDIVRRVILRMSQESDIFGPISSILSTPFVLRTNSVSAM
HEDDTSELQEVARILKDLGVSEVPMKVRKLVVKICDVVTRRAARLAAAGIAGILKKVGRD
GSGGGRRSDKQIMRRTVVAVEGGLYLNYRMFREYMDEALRDILGEDVAQHVVVKAMED
GSSIGSALLLASSQSVQTIPSV*
>*AtHKL3*
MTRKEVVLAVTAATITAVAAGVLMGRWIRRKERRLKHTQRILRKFARECATPVSKLWAVA
DALVADMTASLTAECCGSLNMLVSFTGSLPSGDEKGVHYGVNLRGKELLLLRGTLGGNEE
PISDVQKHEIPIPDDVLNGSFKELCDFISLELVKFLAMNPGGEAEEVKNLGFTLTRSVEQIGS
HSISSIHRKSLANDDDEKVLKDLVNDMNESLETHGLKIRMNTALVDNTIGELAGGRYYHK
DTVAAVSLGMGTNAAYIEQAQEISRWKSAIREPQEIVVSTEWGDFRSCHLPITEFDASLDA
ESLNPGHRIFEKMVSGRYLGEIVRRVLLKMSEESALFGDTLPPKLTIPYILWSPDMAAMHQ
DISEERETVNKKLKEVFGIMDSTLAAREVVVEVCDVVAERAARLAGAGIVGMIKKLGRLE
KKMSIVIVEGGLYDHYRVFRNYLHSSVWEMLGDELSDHVVIEHSHGGSAAGALFLAACG
DGHQDSESK*
>*SvHXK1*
MVAAAAERVVAELREACAAPAARLNEVAAAMEAEMRAGLREEGGSKIKMIISYVDNLPT
GNEEGFFYSLDLGGTNFRVLRVQLAGKEKRVAKRESKEVSIPPHLMSGNASELFGFIASAL
AKVIVDEGRNDVFEDKHRELGFTFSFPVRQTSIASGTLIKWTKAFSIDDAVGEDVVAELRT
AMEKHGVDMRVAALINDTVGTLAAGRYNDEDVVIGVILGTGSNAAYVEEASAIPKFEGEL
PKSGNMVINTEWGNFYSSCLPITEYDQALDEESLNPGEQIFEKLISGMYLGEIVRRVLLKIA
SQSTLFGKVNHTKLKTRFILRTPDISAMHHDETPDLRIVAEKLADNFKIKDTSLETRKMVV
EICDVVTSRSARLAAAGIVGILRKIGRAVPGDERRSVVAIDGGLFEHYTEFRQCLESTLVEL
LGEEASRSVAVKLTKDGSGLGAALIAAAHSQYQH*
>*SvHXK2*
MGKAAAVGTAVVVCAAVGVAVVLARRRRRDAELLGSADADRKRRAAAVIEEVERSLAT
PTALLRSIADAMVTEMERGLRADIHAQLKMLISYVDNLPTGDEHGLFYALDLGGTNFRVL
RVQLGGREKRVVKQQYEEVSIPPHLMVGTSLELFDFIAAALAKFVDTEGEDFHLPEGRQR
ELGFTFSFPVNQTSISSGTLIKWTKGFSVNGMVGEDVVSELSKAMERQGLDMKVTALVND
TVGTLAGGRYMDNDVVAAVILGTGTNAAYVEHANAIPKWTGLLPKSGNMVINTEWGSF
KSDKLPLSEYDKAMDFESLNPGEQIYEKLISGMYLGEIVRRILLKLAHDASLFGDVVPSKL
EQPFVLRTPDMSAMHHDSSHDLKILGAKLKDIVGVADTSLEVRYITRHICDLVAERGARLA
AAGIYSILKKIGRDKVPSNGSKMPRTVIALDGGLYEHYKKFSSCVETTLTDLLGEEASSSV
VAKLANDGSGIGAALLAASHSQYAEAD*
>*SvHXK3*
MGRAQWLSVAVGCAAAVTCAVAAALVSRRAAARCRWNRAVEVVRGFEEGCATPTERLQ
RVVNSLSVEMFAGLASEGASKVRMLLTCVDELPDGSEEGIYYAIDLGGTSFRVMKLELGP
GSMVINKKVEHQPIPEELTKGASEDLFNLIASALKNFIEREGGKDEGRALGFTFSFPVRQISI
SSGSLIRWTKEFSIEEAVGMDVAQCLNEALVRNGLNLQVTALVNNAVGTLAMGHYYDED

TVAAVIIGAGTNASYIERTATITKCQGLLTNSDITVVNVEWGSFRPPQIPLTPYDICFNVEKD
RNHYDQAFEKMISGVYLGEIARLVLQGMAQESDVFGSSVDFLSTPFIFSTPCLAAIREDDSP
DLRVVGRVLEEQLKIQDVPLKTRRLVVRICDIVTRRAARLAAAGIVAVLQKIGRDGTLCGT
SMVRKIRGKPKRSVVAIEGGLYQGYSVFREYLNEAVDEILGDEIASTVSLRVMEEGSGIGA
ALLAASYSSTRQNSA*

>*SvHXK4*

MAAAEAVAMAEQVVADLREKCETPPELLREVASAMAHEMGAGLEKDGGSRVNMLLSYV
DKLPTGREEGLFYGLDLGGTNFRVLRVQLGGIEKHVVNRESREVSIPPQLMSGSSSELFGFI
ASELAKFVADEEKCANVLSNGKKRELGFTFSFPVKQRSVASGTLVKWTKAFSIDDAVGED
VVAELQTAMEKQGLDMHVAALINDAVGTLAGARYYDRDVVAGVIFGTGTNAAYVEKAN
SIPKWEGELPNSGDMVINMEWGNFCSSHLPVTEYDRELDKESLNPGEQIYEKLMSGMYL
GEIVRRVLLKMSLQSSIFGNIDHTKLKTHFLLRTPHISAMHHDETPDLKIVAEKLEENLEITG
TSLETRKLVVEICDIVARRAARLAAAGLAGILKKLGRDGNAQEQRSVIAIDGGLFEHYTKF
RECLEITLGELLAEYASKSVAVKHADDGSGIGAALIAASQSQYRNVE*

>*SvHXK5*

MGKGAVVGTAVVVCAAAAAAVGVAVVVSRRRRRREAENERKRKAAAVIEEVEQAFSTP
TALLRGIADAMVVEMERGLRADPHAPLKMLISYVDNLPTGDEHGLFYALDLGGTNFRVIR
VQLGGREKRVVKQQYEEVSIPPHLMVGTSTELFDFIAAELEKFVRTEGEDFHLPDGKQRE
LGFTFSFPVHQTSISSGTLIKWTKGFSINGTVGEDVVAELSRAMERQGLDMKVTALVNDTV
GTLAGGRYVDNDVAAAVILGTGTNAAYVEHANAIPKWNGLLPRSGNMVINMEWGNFRS
DKLPSSEYDKALDFESLNPGEQIYEKMISGMYLGEIVRRILLKLAHDASLFGDVVPPKLEQ
LFVLRTPDMSAMHHDTSHDLKHLGAKLKDILGVPDTSLEARYITLHVCDLVAERGARLAA
AGIYGILKKLGKDKLPNDFSQQRTVVAMDGGLYEHYKKFSACLEATLTDLLGEEAASSVV
VKLANDGSGIGAALLAASHSQYAEAA*

>*SvHXK6*

MGRVGLGVAAGCAAATCAIAAALVARRASARARWRRAVALLREFEEGCATPPARLRQVV
DAMVVEMHAGLASDGGSKLKMLLTFVDALPTGNEEGIYYAIDLGGTNFRALRVEVGSGS
VVTSRKVELPIPEELTKGTIEELFNFVAIALKDFVEREDGKDEKRALGFTFSFPVRQTSVSS
GSLIRWTKGFSIENAVGKDVAQCLNEALAMSGLNVRVTALVNDTVGTLALGHYHDEDTV
AAVIIGAGTNACYIERTDAIIKCQGLLTNSGGMVVNMEWGNFWSSHLPRTPYDISLDDETQ
NRNDQGFEKMISGIYLGEIARLVLHRMALESDVFGDAADNLSTPFTLSTPLLAAIREDDSP
DLSEVRRILQEHLKIPDTPLKTRRLVVKVCDIVTRRAARLAAAGIVGILKKLGRDGSGVAS
SGRTRGQPRRSVVAIEGGLYQGYPVFREYLDEALVEILGEEVARTVALRVTEDGSGVGAAL
LAAVYSSNRQQGSI*

>*SbHXK1*

MRKPAALVASTVVFAAAAVAAVMARQRLREARRWARAAAMLRDLQERCAAPAERLRQV
ADAMAAEMRAGLASNDSEGESGSTVLLKMLVTYVDSLPSGGEKGLFYALDLGGTNFRVL
RIQFGGKEQRIVKQESKGVSIPQHLMSRGSNELFDFIAAALAKFVASEGEDFHLPEGMQRQ
LGFTFSFPVNQNSVASGTLIKWTKGFAIDEMVGKDVVAELNKAIERQGIDMKITALVNDTV
GTLAAGRYVDNDTVAAVILGTGTNAAYIEHMNSIPKWCGPPPESDDMVINMEWGNFRSS
HLPLTEFDVALDAESLNPGEQIYEKLISGMYMGEIVRRVLLKMAQDASLFADNVPPKLEIP
YILRTYHVLMMHQDTSPDLRTVGINLKEIFGIENTCCKTRKLVVDVCEAVATRGARLAAA
GIHGILKKLGRDIANPDKQKTVIAVDGGVYKYYTFFAQCMESTLRDLLGEEVASSVVIKLA
EDGSGTGAALLAASYSQRFGAMDD*

>*SbHXK2*
MGKGAVVGTAVVVGAAAAAAVGVAVVSLSRRRRRRREAEDERKRKAAAVIEEVEQRFST
PTALLRGIADAMVEEMERGLRAEPHAPLKMLISYVDNLPTGDEQGLFYALDLGGTNFRVI
RVQLGGRDKRVVKQQYEEVSIPPHLMVGTSTELFDFIAAELEKFVRTEGEDFHLPNGKQR
ELGFTFSFPVHQTSISSGTLIKWTKGFSINGTVGEDVVAELSRAMERQGLDMKVAALVNDT
VGTLAGGRYADNDVVTAVILGTGTNAAYVEHANAIPKWTGLLPRSGNMVINMEWGNFRS
DKLPMSEYDKSLDFESLNPGEQIYEKMISGMYLGEIVRRILLKLAHDASLFGDVVPPKLEQ
LFILRTPDMSAMHHDTSHDLKHLGAKLKDILGVPDTSLEARYITLHVCDLVAERGARLAA
AGIYGILKKLGKDKLLSDFSQQRTVVAIDGGLYEHYKKFSACLEATLTDLLGEEVASSVVV
KLANDGSGIGAALLAASHSQYAEVA*
>*SbHXK3*
MGKAVVVGTAVVACAAVGVALALAHRRRKRDAELLGSAEAERKRRAAAVIEDVESSLAT
PTALLRSIADAMVTEMERGLRGDIHSQLKMLISYVDNLPTGDEHGLFYALDLGGTNFRVL
RVQLGGREKRVVKQQYEEVSIPPHLMVGTSMELFDFIAAALAKFVDTEGEDFHLPEGRLR
ELGFTFSFPVNQTSISSGTLIKWTKGFSINGTVGEDVVSELSRAMERQGLDMKVTALVNDT
VGTLAGGRYMDNDVVAAVILGTGTNAAYVEHANAIPKWTGLLPKSGNMVINTEWGSFKS
DKLPLSEYDKAMDFESLNPGEQIYEKMISGMYLGEIVRRILLKLAHDASLFGDVVPSKLE
QPFILRTPDMSAMHHDSSHDLKTLGAKLKDIVGVADTSLEVRYITRHICDLVAERGARLAA
AGIYSILKKIGRDKVPSSGGKMPRTVVALDGGLYEHYKKFSSCVEATLTDLLGEEASSSVV
AKLANDGSGIGAALLAASHSQYGESH*
>*SbHXK4*
MATAALAMAEQVVADLRAKCEAPPPMLREVAAEMAREMGAGLEKEGGSRVKMLLSYV
DKLPTGGEEGLFYGLDLGGTNFRVLKVELGGNEKHVVDRDSREVIIPPHLMSGSSSELFGF
IASELAKFVVDDEKFINVLNGKKRELGFTFSFPVKQRSVASGTLVKWTKAFSINDAVGEDV
VAKLQTAMEKQGLDMHVAALINDAVGTLAGARYYDKDVVAGVIFGTGTNAAYVEKANA
IPKWEGELPNSGDMVINMEWGNFCSAYLPITEYDQELDKESLNPGEQIYEKLTSGMYLGEI
VRRVLLKISLQSAIFGNIDHTKLETPFLLRTPHISAMHHDETPDLKIVAKKLEENLEITGASL
EARKLVVEICDIVATRAARLAAAGLAGILMKLGRDCSVEDQRSVIAIDGGLFEHYTKFRQC
LETTLGELLGDEASKAVAVKHADDGSGIGAALIAASQSLYKNDLVAVKHADDKHADDKH
EDADDKHEDDGKGVKHADDGSEIGAALIAASQSQ*
>*SbHXK5*
MVAAAAERVVAELREACAAPAARLNEVAAAMEAEMRAGLREEGGSKIKMIISYVDNLPT
GNEEGVFYALDLGGTNFRVLRVHLAGKDKRVAKRESKEVSIPPHLMSGNASELFGFIASAL
AKYIASEEGHSNVFDDDKQRELGFTFSFPVRQTSIASGTLIKWTKAFSIDDAVGEDVVAEL
QTAMEKQGVDMRVAALINDTVGTLAAGRYNDEDVVIGVILGTGSNAAYVEEASAIPKLE
GELPKSGNMVINTEWGNFDSSCLPITEYDEALDEESLNPGEQIFEKLISGMYLGEIVRRVLL
KIASQSSIFGNVSHTLLKTRFILRTPDISAMHHDDTPDLRIVAQKLADNLKIMDTSLDTRKM
VVEICDIVTSRSARLAAAGIVGILRKIGRAVPGDERRSVVAIDGGLFEHYAEFRKCLESTLV
ELLGEEASKSLVVKLTKDGSGLGAALIAAAQSQ*
>*SbHXK6*
MGRVGLGVAAGCAAATCAIAAALVARRASARARWRRAVALLREFEEGCATPTPRLRQVV
DAMVVEMHAGLASDGGSKLKMLLTFVDALPAGGGAVGRAFPTYLAPNAACIERCIPQW
HCGLGRSLSDGNHCSGRHHLLLSKFIVLFWVAALATAYNDSRTICVTDGIHIDLVGLLFFCF
CHINEQGTYYSIDLGGTNFRVLRVEVGAVSVVTSREVKLPIPEELTKGTIEELFNFVAMTLK

EFVETEDVKDEQRALGFTFSFPVRQTSVSSGSLIRWTKGFLIEDAVGKDVAQCLNEALARS
GLNVRVTALVNDTVGTLALGHYYDEDTVAAVIIGAGTNACYIERTDAIIKCQGLLTNSGG
MVVNMEWGNFWSSHLPRTPYDISLDDETQNRNDQGFEKMISGIYLGEIARLVLHRMALE
SDVFGDAADHLSTPFTLSTPLLAAIRKDDSPDLSEVRRILQEHLKIMDTPLKTRRLVVKVC
DIVTRRAARLAAAGIVGILKKLGRDGSGVASSGRTRGQLRRTVVAIEGGLYEGYPVFREYL
DEALVEILGEEVAQTVALRVTEDGSGAGAALLAAVHSSNRQQGSI*

>*SbHXK7*

MGKAQWLSVALGCVAAVTCAVATTLVARRAVARYRWSRAVAVVRGFEEDCATPTERLQRI
VNSLSVEMFAGLASEGASKVRMLLTCVDALPDGSEEGIYYSVDLGGTSFRVMKLELGSGS
MVINKKVEHQPIPEDLTKGTSEDLFNLIALALKNFIEREGGGDEGRALGFTFSFPVRQVSIS
SGSLIRWTKEFSIEEAVGRDVAQCLNEALVRNGLNLQVTALVNNAVGTLAMGHYYDEDT
VAAVIIGAGTNASYIERNAAIPKSQGLLTNSDITVVNVEWGSFRSPQIPLTPYDICSSEAERN
HYDQAFEKMISGVYLGEIARLVFQRMAQESDLFGSFVNCLSTPFILSTPSLAAIREDDSPDL
RVVGKVLEEHLKIQDVPLKTRRLVVRICDIVTRRAARLAAAGIVAILQKIGRDGTLCGTTF
VRKIRGEAKRSVVAIEGGLYQGYSVFREYLNEAVDEILGEELAPTVSLRVMEEGSGIGAAL
LAASYSSTRKNFV*
