# Supplementary material for: Genomic Analysis of Hexokinase Genes in Foxtail Millet (Setaria italica): Haplotypes and Expression Patterns Under Abiotic Stresses
Source: Int J Mol Sci. 2025 Feb 24;26(5):1962. doi: 10.3390/ijms26051962 (PMC11900577; doi:10.3390/ijms26051962)
Supplement: Supplementary file 1 [file ijms-26-01962-s001.zip › Table S2 The conserved motifs of the genes in the HXKs gene family are determined by MEME..pdf]

Table S2. The conserved motifs of the genes in the *HXKs* gene family are determined by MEME.

Motif name	Motif logo
Motif 1	VGEDVVAELNEAMERQGLDMRVTALVNDTVGTLAGGRYYDEDVVAAVILGTGTNAAYVERABAIPK
Motif 2	LKTPFILRTPDMSAMHHDTSPDLKIVGAKLKDILGIPDTSLETRKIVVEICDIVAERGARLAAAGIYGILKKJGRDKTP
Motif 3	RELGFTFSFPVRQTSISSGTLIKWTKGFSIDDA
Motif 4	SEGGSKLKMLJSYVDNLPTGBEEGLFYALDLGGTNFRVLRVQL
Motif 5	LNPGEQIYEKMISGMYLGEIVRRVLLKMALDSSJFGDVVPT
Motif 6	TVVAIDGGLYEHYTKFRECLEETLVELLGEEASSSVVVKLANDGSGIGAALLAASHS
Motif 7	KRVVKQZSEEVSIPPHLMSGTSEELFDFIASALAKFVATEG
Motif 8	GLLPNSGNMVINMEWGNFRSSHLPLTEYD
Motif 9	ELEEACATPTALLRQVADAMAVEMEAGLA
Motif10	IPDTPLKTRRLVVKVCDIVTRRAARLAAAGIVGILKKJGRD
