# Supplementary material for: Genomic Analysis of Hexokinase Genes in Foxtail Millet (Setaria italica): Haplotypes and Expression Patterns Under Abiotic Stresses
Source: Int J Mol Sci. 2025 Feb 24;26(5):1962. doi: 10.3390/ijms26051962 (PMC11900577; doi:10.3390/ijms26051962)
Supplement: Supplementary file 1 [file ijms-26-01962-s001.zip › Table S3 qRT-PCR Prime.pdf]

Table S3 Primers used in the study

Gene ID	Sense primers (5'-3')	Anti-sense primers (5'-3')	Application
SiHXK1	GTAGATACAGAGATGAGGAT	CAGGATGAGTAGGAGGTTACC	For qRT-PCR to detect expression level
SiHXK2	AACTACATCATCAGAGAA	AACTACATCGTTAATCATAT	For qRT-PCR to detect expression level
SiHXK3	GCATTATTAGTAGGTAGGAG	CATAAGGAGGTTAGTATGAT	For qRT-PCR to detect expression level
SiHXK4	CAAGATACTATGACAGAGATG	ATATTAGTGAGCAGGAAGAT	For qRT-PCR to detect expression level
SiHXK5	CGAAGCAGGATACATTATCTC	ATATAAGCCACCATCCATAG	For qRT-PCR to detect expression level
SiHXK6	CAAGAACTCCTTAGTGACATC	ATAGATTAGACGAGCAGCATC	For qRT-PCR to detect expression level
Actin	GACATGGCTGGTCGTGAT	AGTGGTGGAGAAGGTGTAAC	Internal standard
SiHXK5	CACTCGGACTTAAGACTAGTAT GGGGAGGCGGCGTC	AGCTCCGGACTTAAGACTTGTA TGGAACTTGTGCTCTATTCGA	For subcellular localization
SiHXK6	CACTGGGTACCTGCAACTAGTA TGGGGAGGGCGGCGTC	AGCTCCGGACTTAAGACTAGTT GCAGCTTCAGCATAGGGA	For subcellular localization
